# Preliminary Evidence of “Other-Race Effect”-Like Behavior Induced by Cathodal-tDCS over the Right Occipital Cortex, in the Absence of Overall Effects on Face/Object Processing

**DOI:** 10.3389/fnins.2017.00661

**Published:** 2017-11-30

**Authors:** Andrea I. Costantino, Matilde Titoni, Francesco Bossi, Isabella Premoli, Michael A. Nitsche, Davide Rivolta

**Affiliations:** ^1^School of Psychology, University of East London, London, United Kingdom; ^2^Department of Psychology, University of Milano-Bicocca, Milan, Italy; ^3^Department of Basic and Clinical Neuroscience, Institute of Psychiatry, Psychology and Neuroscience, King's College, London, United Kingdom; ^4^Department of Psychology and Neuroscience, Leibniz Research Center for Working Environment and Human Factors, Dortmund, Germany; ^5^Department of Neurology, University Medical Hospital Bergmannsheil, Bochum, Germany; ^6^Department of Education, Psychology and Communication, University of Bari Aldo Moro, Bari, Italy

**Keywords:** face processing, object processing, tDCS, other-race effect, neuromodulation

## Abstract

Neuromodulation techniques such as tDCS have provided important insight into the neurophysiological mechanisms that mediate cognition. Albeit anodal tDCS (a-tDCS) often enhances cognitive skills, the role of cathodal tDCS (c-tDCS) in visual cognition is largely unexplored and inconclusive. Here, in a single-blind, sham-controlled study, we investigated the offline effects of 1.5 mA c-tDCS over the right occipital cortex of 86 participants on four tasks assessing perception and memory of both faces and objects. Results demonstrated that c-tDCS does not overall affect performance on the four tasks. However, *post-hoc* exploratory analysis on participants' race (Caucasian vs. non-Caucasians), showed a “face-specific” performance decrease (≈10%) in non-Caucasian participants *only*. This preliminary evidence suggests that c-tDCS can induce “other-race effect (ORE)-like” behavior in non-Caucasian participants that did not show any ORE before stimulation (and in case of sham stimulation). Our results add relevant information about the breadth of cognitive processes and visual stimuli that can be modulated by c-tDCS, about the design of effective neuromodulation protocols, and have important implications for the potential neurophysiological bases of ORE.

## Introduction

Faces represent the stimuli we rely the most for social interaction, and their processing is mediated by dedicated cognitive and neurophysiological signatures (Kanwisher, 2010; Rivolta et al., 2014b). Since deficits in face perception characterize various neurodevelopmental conditions such as autism (Tang et al., 2015), schizophrenia (Rivolta et al., 2014a), and congenital prosopagnosia (i.e., the lifelong inability in recognizing people by their faces; Rivolta et al., 2012a), it is important to find techniques/methodologies that help to ameliorate face-processing skills.

In this context, a critical role might be played by transcranial direct current stimulation (tDCS; Nitsche and Paulus, 2000), which consists in delivering a small current (1–2 mA) through two electrodes (i.e., a “target” and a “return”) placed over the human scalp (Nitsche et al., 2003b). tDCS can be administered in *anodal* or *cathodal* modality, referring to the polarity of the current delivered by the target electrode (Stagg and Nitsche, 2011). Studies in the human motor cortex indicate that anodal-tDCS (a-tDCS) causes subthreshold depolarization (i.e., increased excitability), whereas cathodal-tDCS (c-tDCS) causes subthreshold hyperpolarization (i.e., decreased excitability) of critical neuronal compartments of the target area (Creutzfeldt et al., 1962). A few minutes of stimulation can induce aftereffects, which reflect calcium (Ca+)-dependent plastic changes mediated by the N-methyl-D-aspartate receptor (NMDA-R), thus resembling long-term-potentiation (LTP)- and long-term-depression (LTD)- like plasticity to a certain extent (Liebetanz et al., 2002; Nitsche et al., 2003a). This has led to the (often incorrect) inference that, at least in the cognitive domain, a-tDCS enhances performance, whereas c-tDCS decreases it (Bestmann et al., 2015). Evidence, however, points toward a more complex picture; while anodal stimulation usually shows cognitive enhancement, cathodal effects are less clear (Jacobson et al., 2012). Albeit recent studies showed improved face-processing skills after occipital (Barbieri et al., 2016) and fusiform (Brunyé et al., 2017) a-tDCS, it is still unknown whether c-tDCS would lead to an opposite outcome (i.e., decreased performance), a null effect or even a cognitive enhancement. This will have important implications for the design of rehabilitative protocols, and to our understanding of the neurophysiological mechanisms that mediate human visual cognition. Thus, the main aim of the current study is to assess the effects of a single session of c-tDCS on face and object processing. Objects have been included as a “control” condition to ascertain whether potential effects of c-tDCS are face-specific. Given that face and object selective brain areas are closely neighbored on the lateral surface of the right occipital lobe (Dilks et al., 2013; Rivolta et al., 2014b), we expect an affect (if present) on both categories (Barbieri et al., 2016).

## Methods

### Participants

Eighty-six healthy participants (*M* = 26.65 years, range 19–49; 41 male, 45 female; 48 Caucasians, 38 non-Caucasians) participated in this single-blind, sham-controlled study (Table 1). Participants were selected if they fulfilled the criteria of: (1) no history or evidence of chronic or residual neurological disease (2) no metal implants in neck or head area or pacemakers (3) no intracerebral ischemia or history of bleeding, epilepsy, head injury (4) no serious medical conditions, pregnancy or psychiatric illness (5) no alcohol, drug addiction or participation in a study involving drug intake within the last month, (6) normal or corrected-to-normal vision, and (7) at least the last 5 years spent living in the UK (to exclude the other race effect, ORE, at baseline) (Goldstein and Chance, 1985; Tanaka et al., 2004; Michel et al., 2006; McKone et al., 2007). The study was performed in accordance with the Code of Ethics of the World Medical Association for experiments involving humans, and approved by the ethical committees of the University of East London (UEL). Before each session, participants were asked to read and sign both a written information letter about the purpose and the procedure of the study and an informed consent form.

**Table 1:** Demographic features of the sample (sham and c-tDCS) indicating the sample size (N), the ratio between males and females (M/F), and age (mean and *SD*).

	**Sham**	**c-tDCS**
N	43	43
M/F	21/22	20/23
Age	26.05 (5.88)	27.26 (7.14)

### Experimental design

Participants were assigned to one of the two experimental groups (“Sham” and “Cathodal”) (see next section for the description of the stimulation protocol). As suggested by previous works (Inghilleri et al., 2004; Fertonani et al., 2011), given that progesterone and estrogen levels seem to influence cortical excitability, we recruited female subjects only during the follicular phase of their menstrual cycle—i.e., when their hormonal levels least likely influence neuromodulation effects.

**Figure 1:**
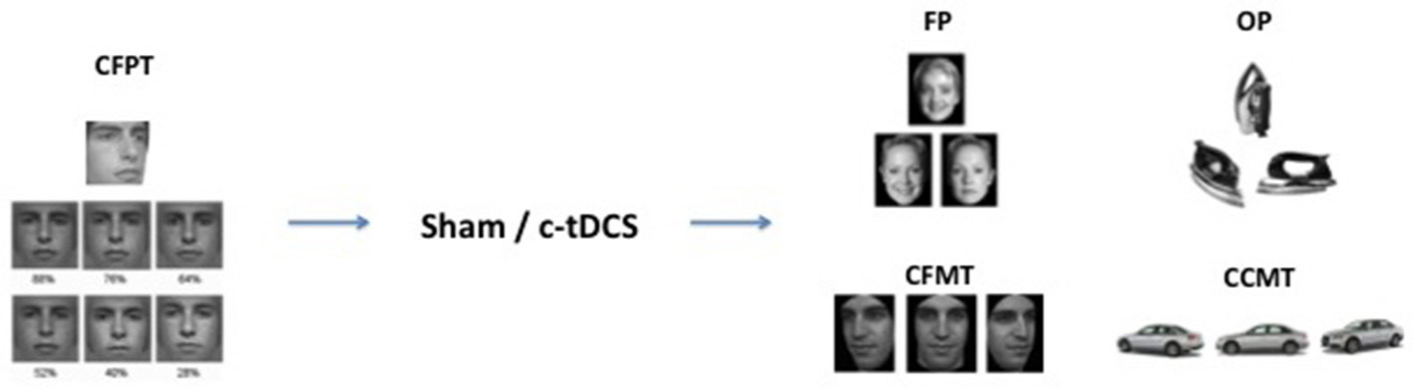
Experimental design and examples of trial stimuli. The Cambridge Face Perception Task (CFPT) was administered before tDCS (sham or c-tDCS). After 20 min of stimulation participants completed four tasks: face perception task (FP), object perception task (OP), Cambridge Face Memory Test (CFMT), and Cambridge Car Memory Test (CCMT).

Following our previous study, a baseline measure was recorded to explore unexpected differences in face recognition abilities between the two groups. Each subject thus completed the Cambridge Face Perception Task (CFPT) (Duchaine et al., 2007) *before* the tDCS was set up. In the CFPT, subjects had to sort a set of six faces from the most familiar to the least one according to a target face. Each face had a specific percentage of the target face (from 88 to 28%). After the CFPT and the c-tDCS application, both groups performed a set of four tasks in counterbalanced order: The Face Perception task (FP), the Object Perception task (OP), the Cambridge Face Memory Task (CFMT), and the Cambridge Car Memory Task (CCMT) (see Barbieri et al., 2016 for the same design) (Figure 1). In the Face Perception task (FP), a set of three gray-scale, unfamiliar faces were presented to the subject in each trial. In each set, two faces belonged to the same person and were presented from two different angles, while the third face looked similar to the others but belonged to a different person. Subjects were required, using the Up, Left, and Right arrow keys on the keyboard, to identify the “odd one,” that is the face with a different identity. The task was composed by 81 trials, and each trial had a time limit of 4 s. The Object Perception task (OP) had the same structure as the FP, but involved objects recognition rather than faces. Stimuli from the FP and OP were taken from previously published studies (Barense et al., 2011).

The Cambridge Face Memory Task (CFMT) (Duchaine and Nakayama, 2006) is a memory task using unfamiliar faces as stimuli. The CFMT requires participants to memorize a set of six Caucasian male faces after a brief exposure. After the practice, subjects were asked to identify the familiar faces between three for each trial, in three different conditions: (1) faces with the same light and angulation condition; (2) faces with different light/angulation condition; (3) faces with different levels of noise. In this final step, different levels of Gaussian noise were added to each trial, with the purpose of engaging specific face processing mechanisms. The Cambridge Car Memory Task (CCMT) (Dennett et al., 2012) has the same structure as the CFMT, but uses car instead of face stimuli. All tasks were run on Windows, and were administered on a DELL desktop computer with a 17-inch monitor with a resolution of 1,152 × 864 pixels.

### Transcranial direct current stimulation (tDCS)

tDCS was delivered by a Neuroelectrics® (Barcelona, Spain) stimulator via a pair of surface sponge electrodes (25 cm^2^) soaked in saline solution (0.9% NaCl) and applied to the cathode/target and the anode/return areas (respectively PO8 and Fp1 according to the 10–20 EEG system). Stimulation parameters and timing were identical to those used in Barbieri et al. (2016). In the cathodal condition (c-tDCS) we administered a constant current of 1.5 mA (current density: 0.080 mA/cm^2^) for 20 min, before (i.e., offline) the four main tasks. In the sham condition stimulation was only maintained for the first and last 10 s to evoke the sensation of being stimulated, without causing neurophysiological changes that may influence performance. During the stimulation participants were comfortably placed on a chair and asked not to interact with the experimenter.

**Figure 2:**
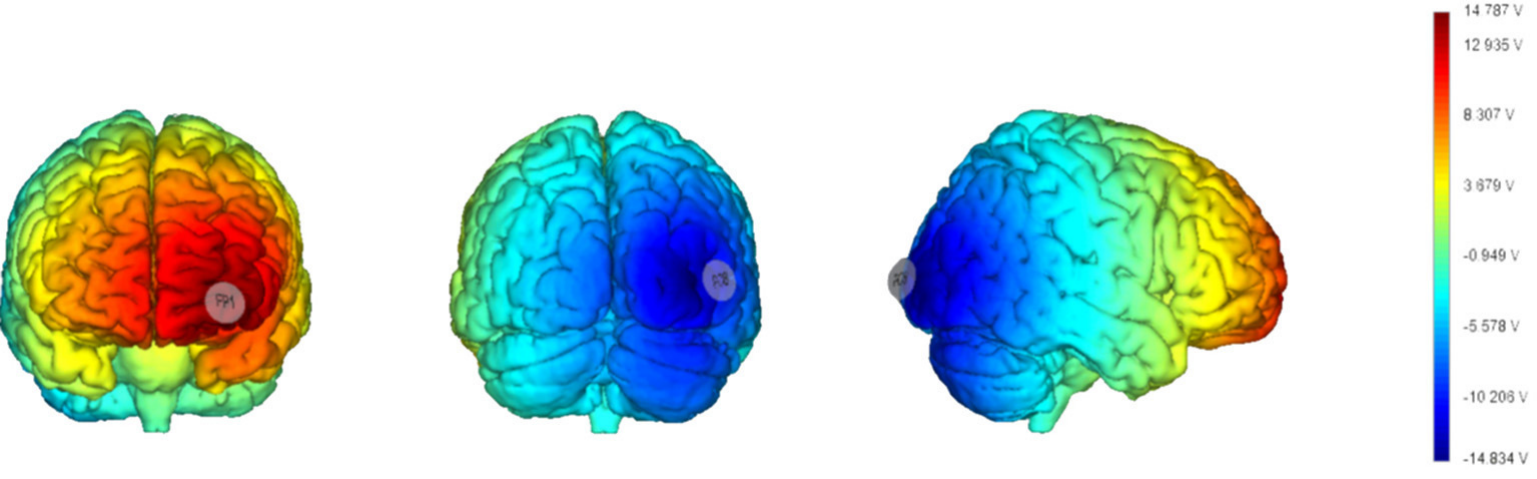
Current distribution estimated based on a template brain (**left**: frontal cortex; **middle**: occipital cortex; **right**: right hemisphere) with a realistic brain finite element (FEM) model (electrodes size: 25 cm^2^). The model has been generated using StimViewer (Neuroelectrics®).

As in Barbieri et al. (2016) we chose a bipolar-non-balanced montage (Nasseri et al., 2015), with PO8 as the target site, because of it is involved in the generation of face-sensitive neurophysiological features (i.e., N170; Rossion et al., 2000; Negrini et al., 2017), and the prominence of the right hemisphere for face processing (Kanwisher, 2010; Rivolta et al., 2012b). The return electrode was placed over Fp1 since the left frontopolar cortex has no known relevant role in visual cognition, and to maximize the distance between the target and return electrodes in order to increase current density in depth (Rockstroh et al., 1989; Figure 2).

### Statistical analyses

To test for unexpected baseline differences between groups, a *t*-test was performed on participants' CFPT accuracy scores. To ascertain whether c-tDCS affects visual cognition, accuracy and RTs data were analyzed with a mixed 2 × 4 ANOVA, with the between factor “stimulation” (Sham vs. Cathodal) and the within-subject factor “task” (FP, OP, CFMT, CCMT) (we refer to this as analysis 1). A second, “exploratory analysis” (Analysis 2), was carried considering participants' race (see the rationale of the analysis in the paragraph below). We conducted a mixed 2 × 2 × 4 ANOVA on accuracy and RTs, with “race” (Caucasian, Non-Caucasian) and “condition” (Sham, Cathodal) as between-subjects factors, and “task” (FP, OP, CFMT, CCMT) as a within-subject factor. In order to explore significant interactions, *post-hoc* comparisons (Bonferroni-corrected) were performed. To ascertain whether potential race-specific effects of c-tDCS were not due to non-controlled variables, a Chi-squared test was run to check whether the distribution of males and females across conditions was similar. In addition, a 2 × 2 ANOVA with factors condition (sham vs. c-tDCS) and race (Caucasians vs. non-Caucasians) was conducted to test whether the age of participants did not differ across the four conditions (we refer to this as analysis 2). Similarly to analysis 1, we checked whether the groups did not differ in baseline (i.e., CFPT) by using a mixed 2 × 2 ANOVA with factors Condition (sham vs. c-tDCS) and race (Caucasians vs. non-Caucasians). All analyses were conducted using SPSS Statistic Software (IBM Corp. Released 2013. IBM SPSS Statistics for Windows, Version 23.0. Armonk, NY: IBM Corp).

#### Rationale for the exploratory analysis (analysis 2)

It is known that face-processing skills are influenced by the race (Chance et al., 1982), and even ethnicity, (McKone et al., 2011) of the face stimuli. In particular, people are better at recognizing faces of their own race; and this is known as the “other-race effect” (ORE) (Meissner and Brigham, 2001). Albeit the ORE likely disappears (or it is at least reduced) after (even short) exposure to the other-race (Sangrigoli and de Schonen, 2004; Michel et al., 2006; McKone et al., 2007), the effects of neuromodulation on perception of same- and other- race faces still remains unexplored. In addition, given that the ORE is generally mediated by visual exposure (i.e., expertise; Wan et al., 2015), and since visual learning is likely driven by plasticity effects (Ramoa et al., 2001), it is possible that c-tDCS, by inducing long-term depression (LTD)-like phenomena (Nitsche et al., 2003a), might disproportionally affect the perception and memory of other-race faces. Previous research using neuromodulation, even when conducted in highly multicultural countries such as Australia (Willis et al., 2015) or UK (Romanska et al., 2015), has not specifically considered the race of participants and how it interacts with the race of the face stimuli adopted in the experiments. As such, we here ascertained whether a single session of c-tDCS differentially affects Caucasian and non-Caucasian individuals, even when baseline performance does not distinguish the two races, *thus excluding ORE before neuromodulation*. In addition, we assessed whether the c-tDCS effect was face-specific or not.

## Results

### Analysis 1

**Figure 3:**
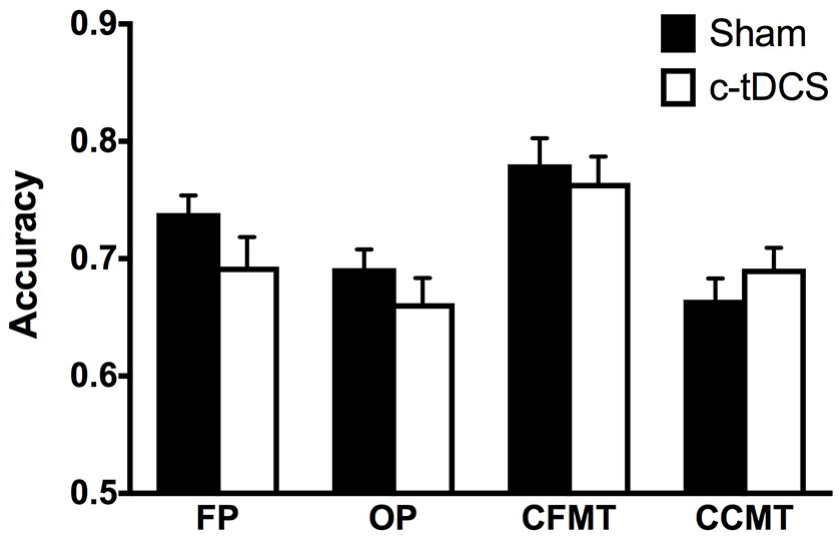
Sham (black) and c-tDCS (white) accuracy results on the face perception task (FP), object perception task (OP), Cambridge Face Memory Test (CFMT), and Cambridge Car Memory Test (CCMT). Error bars represent the SEM.

There were no statistically significant differences in CFPT (i.e., baseline) performance between Sham (mean = 39.40, *SD* = 15.58) and c-tDCS (mean = 44.42, *SD* = 20.05) groups [*t*_(79.18)_ = −1.3, *p* = 0.20]. Results of the mixed 2 × 4 ANOVA showed no significant effect of condition [*F*_(1, 84)_ = 0.94, *p* = 0.34, η^2^_*p*_ = 0.011] and no Condition x Task interaction [*F*_(2.7, 231.2)_ = 1.49, *p* = 0.22, η^2^_*p*_ = 0.017], thus indicating that, overall, c-tDCS did not affect performance on tasks assessing face and object processing (Figure 3).

### Analysis 2 (exploratory analysis)

Analysis of CFPT (i.e., baseline) showed no main effect of condition [*F*_(1, 82)_ = 2.07, *p* = 0.15, η^2^ = 0.025], no main effect of race [*F*_(1, 82)_ = 2.92, *p* = 0.09, η^2^ = 0.034] and no condition × race interaction [*F*_(1, 82)_ = 1.29, *p* = 0.26, η^2^ = 0.015], thus suggesting that the four groups did not show baseline differences in their face perception abilities (see also Table 2 for the description of the four groups).

**Table 2:** Demographic features of the sample indicating the sample size (N), the ratio between males and females (M/F), and age (mean and *SD*).

	**Caucasians**		**Non-Caucasians**	
	**Sham**	**c-tDCS**	**Sham**	**c-tDCS**
N	24	24	19	19
M/F	11/13	7/17	10/9	13/6
Age	28.25 (6.76)	27.50 (7.44)	23.26 (2.77)	26.95 (6.95)

**Figure 4:**
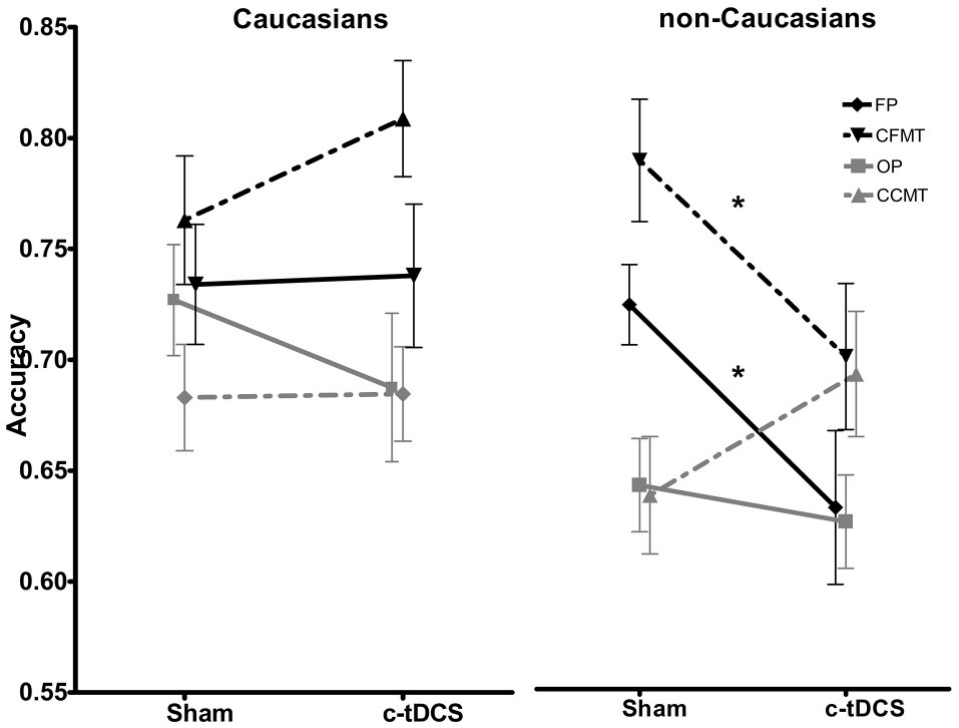
Sham and c-tDCS accuracy scores for the face perception task (FP), object perception task (OP), Cambridge Face Memory Test (CFMT), and Cambridge Car Memory Test (CCMT) are shown for Caucasian **(left)** and non-Caucasian **(right)** participants (^*^p < 0.05). Error bars represent the SEM.

Results of the 2 × 2 × 4 ANOVA on accuracy scores revealed statistically significant main effects of task [*F*_(3, 246)_ = 14.35, *p* < 0.001, η^2^ = 0.139] (FP: mean = 0.72, *SD* = 0.14; CFMT: mean = 0.77, *SD* = 0.15; OP: mean = 0.68, *SD* = 0.12; CCMT: mean = 0.68, *SD* = 0.12), and race [*F*_(1, 82)_ = 5.29, *p* = 0.02, η^2^ = 0.059], with Caucasians (Mean = 0.73; *SD* = 0.13) performing overall better in the four behavioral tasks than non-Caucasians (Mean = 0.69; *SD* = 0.13). Crucially, a statistically significant race × task × condition interaction [*F*_(3, 246)_ = 3.78, *p* = 0.01, η^2^ = 0.037] showed that, in non-Caucasians *only*, c-tDCS caused a performance decrease on FP (Sham: 73.4%, *SD* = 0.08; c-tDCS: 63.7%, *SD* = 0.16; *p* = 0.027) and CFMT (Sham: 80.3%, *SD* = 0.13; c-tDCS: 70.9%, *SD* = 0.15; *p* = 0.046; Figure 4). No other main effects or interactions reached statistical significance (all Ps > 0.05).

Results of the 2 × 2 × 4 ANOVA on RTs only showed a statistically significant effect of task [*F*_(3, 246)_ = 149.09, *p* < 0.001, η^2^ = 0.645] (FP: mean = 2,008 ms, *SD* = 386; OP: mean = 2,047 ms, *SD* = 391; CFMT: mean = 3,055 ms, *SD* = 910; CCMT: mean = 4,575 ms; *SD* = 1,727). No other main effects or interactions reached statistical significance (all Ps > 0.05).

Results of the chi-squared analysis indicated that males and females were equally distributed across conditions [$\chi_{\left(2\right)}^{2}$ = 2.16, *p* = 0.54], thus making it unlikely that gender distribution would have affected the main interaction result. The age of participants did not differ between sham (*M* = 26.0; *SD* = 5.94) and c-tDCS (*M* = 27.3; *SD* = 7.15) [*F*_(1, 82)_ = 0.81, *p* = 0.37, η^2^ = 0.009). Non-Caucasians (*M* = 25.1; *SD* = 5.54) were, overall, younger than Caucasians (*M* = 28.1; *SD* = 7.02) [*F*_(1, 82)_ = 4.73, *p* = 0.033, η^2^ = 0.052]. Crucially, however, the lack of a statistically significant condition x race interaction [*F*_(1, 82)_ = 3.15, *p* = 0.08, η^2^ = 0.035] suggests that the age of participants in the sham and c-tDCS conditions did not differ between Caucasians and non-Caucasians.

## Discussion

Neuromodulation techniques such as tDCS have provided important insight into the neurophysiological mechanisms that mediate cognition. Albeit a-tDCS often enhances cognitive skills, the role of c-tDCS in visual cognition is largely unexplored and inconclusive (Jacobson et al., 2012). Thus, the main aim of the current study was to investigate in a relatively large cohort of participants (*N* = 86) the effects of a single *offline* session of c-tDCS on face and object processing. Results demonstrated that c-tDCS does not, overall, lead to a decrease in cognitive performance in tasks assessing face/object perception and memory. To ascertain whether c-tDCS differentially affects Caucasian and non-Caucasian participants while processing Caucasian faces and objects, we ran a *post-hoc* analysis considering “race” as a factor. Results, albeit preliminary, demonstrated for the first time that c-tDCS causes a “face-specific” performance decrease (≈10%) in non-Caucasian participants *only*. Crucially, this effect emerges despite participants from the two races had the same baseline face perception abilities (i.e., same CFPT performance), and showed no differences in the “sham” condition. Thus, c-tDCS can induce “ORE-like” behavior in non-Caucasian participants that did not show any ORE before stimulation (and in the sham stimulation condition).

### c-tDCS does not always lead to a performance decrease

Neurophysiological evidence suggests that a-tDCS leads to depolarization (i.e., excitation), whereas c-tDCS leads to hyperpolarization (i.e., inhibition) of critical elements of neuronal tissue (Stagg and Nitsche, 2011). This, by generalization, often marshals to the (incorrect) conclusion that a-tDCS leads to enhanced, whereas c-tDCS to decreased cognitive performance. In fact, despite evidence seems to show enhanced cognitive skills (i.e., working memory; visual cognition) induced by a-tDCS (Fregni et al., 2005; Pirulli et al., 2013; Shin et al., 2015), the effects of c-tDCS are less clear-cut (see Jacobson et al., 2012 for a meta-analysis).

Only few studies investigated the effects obtained by a-tDCS applied over posterior face-sensitive areas during face processing tasks. Anodal stimulations indicate an increased working memory for faces after 1.5 mA a-tDCS of the right fusiform gyrus (Brunyé et al., 2017) and enhanced perception/memory for faces (but also objects) after a-tDCS with 1.5 mA over the right occipital cortex (Barbieri et al., 2016). However, there is also evidence that both anodal and cathodal 1.5 mA stimulation lead to a reduction of the composite face effect (i.e., a marker of holistic face processing; Yang et al., 2014). Here, by adopting the same (offline) experimental set up as in Barbieri et al. (2016), we tested whether c-tDCS would lead to an overall decrease in face identification skills. Results, overall, showed no c-tDCS effects on face and object processing. Thus, in line with previous evidence (Jacobson et al., 2012), the simplistic rule of “cognitive enhancement after a-tDCS” and “cognitive decline after c-tDCS” does not seem to hold, at least for higher visual cognitive processing involving face and object perception/memory. This heterogeneity might be due to methodological differences across studies, such as stimulation intensity, timing of stimulation with respect to a task (i.e., online vs. offline), stimulation duration, individual differences, state dependency and task characteristics (Antal et al., 2004a; Kuo et al., 2008; Pirulli et al., 2013; Bestmann et al., 2015; Fertonani and Miniussi, 2016; Hsu et al., 2016).

### c-tDCS over the right occipito-temporal cortex induces “ore-like” behavior

It is known that the perception of “other-race” faces is harder than the perception of faces belonging to the same race (i.e., other race effect; ORE; Chance et al., 1982). The ORE, which is seen already in few months old infants (Singarajah et al., 2017), is due to limited exposure to faces belonging to different races (Wan et al., 2015), and it can be reduced/eliminated by (even short) exposure to other-race faces (Goldstein and Chance, 1985; Sangrigoli and de Schonen, 2004; McKone et al., 2007; de Heering et al., 2010). The neurophysiological factors mediating the ORE are largely unknown and unexplored, but likely mediated by neural plasticity of the visual system. Thus, in the current study we were also interested to investigate the neurophysiological correlates of the ORE in typical human adults.

Our results demonstrated that, after c-tDCS, face (but not object) perception and memory are selectively impaired in non-Caucasians when exposed to “other-race” (i.e., Caucasian) faces. Crucially, since participants did not show ORE for baseline performance and after sham stimulation, our results demonstrate that c-tDCS may induce an acute “ORE-like” behavior. Given that our non-Caucasian participants lived in an “other-race” country for at least five years (see inclusion criteria), our results are in line with our predictions that their ability to recognize other-race faces is, on average, comparable to Caucasians living in the UK (Sangrigoli and de Schonen, 2004; Tanaka et al., 2004; Michel et al., 2006; McKone et al., 2007). It is known that c-tDCS induces excitability reduction of the stimulated cortex, and this effect resembles some features of LTD (Stagg and Nitsche, 2011). Thus, the provisional evidence we provide for the origin of “ORE-like” behavior in non-Caucasian participants suggests that exposure to “other-race” faces might be mediated by the glutamatergic system, and that this can be (at least temporarily) affected by c-tDCS.

A further aspect that deserves attention is that c-tDCS *selectively* impaired face perception/memory; there was no effect on object processing. This was against our initial hypothesis. In fact, since face- and object- sensitive neurons are closely positioned in the lateral occipital cortex (Pitcher et al., 2009), it is surprising that inhibition of this area of the brain did not cause behavioral impairments in both categories of visual stimuli. It is thus possible that the seen differences are mainly driven by distinctive cognitive and neurophysiological mechanisms that mediate human face and object perception. From the cognitive point of view, it is known that while objects are perceived by means of featural processing (i.e., part-based processing), typical face perception also relies on holistic processing, which refers to the ability to perceive faces as wholes (McKone and Yovel, 2009; Palermo et al., 2011). Given that other-race face perception is generally mediated by weaker holistic processing—albeit this might change after exposure to other-race faces—(Tanaka et al., 2004; Michel et al., 2006; McKone et al., 2007; Rhodes et al., 2009), it is likely that c-tDCS, by targeting holistic processing, causes face-specific impairments in non-Caucasians only. That is, c-tDCS (at least with the parameters we adopted) is not sufficient to cause face-specific deficits in Caucasians because holistic processing for Caucasian faces might be stronger in these participants than non-Caucasians holistic processing for Caucasian faces (Mondloch et al., 2010); and/or because same-race face perception shows stronger functional connectivity across face-sensitive areas (Ding et al., 2014; Zhou et al., 2016).

At the neurophysiological level, face perception induces stronger high frequency (>30 Hz) gamma-band oscillations (GBO) than non-face object and inverted face perception (Tallon-Baudry, 2009; Grützner et al., 2013), thus positing for a critical role of GBO in holistic processing. At the neural level, GBO are generated by a mechanism of “feedback inhibition,” which is mediated by glutamatergic NMDA-R activity on GABA-ergic interneurons (Rivolta et al., 2015). Since c-tDCS, possibly by altering GABA-ergic activity (Stagg and Nitsche, 2011), has been shown to reduce GBO (Antal et al., 2004b), it is likely that the face-specific effect we found in the current study is mediated by c-tDCS-induced GBO reduction. This aspect can be directly tested in future studies by combining tDCS with electroencephalographic (EEG) recordings.

A potential limitation of our preliminary results study is that it cannot be completely excluded that the acute “ORE-like” behavior we showed is not site-specific (i.e., due to an effect of c-tDCS on the right occipital cortex), but caused by a general and non-specific effect of stimulation. This however is very unlikely, because the same paradigm, albeit with inverted polarity (i.e., a-tDCS), has been adopted before (Barbieri et al., 2016), and resulted in performance enhancement after stimulation of the right occipital lobe only; this effect was absent after sensory-motor cortex stimulation, thus highlighting the site-specificity of the effect. However, a further active c-tDCS control stimulation condition would be advisable to definitely exclude this possibility. A further aspect to consider is that since baseline differences in task performance might lead to different c-tDCS outcomes (Romei et al., 2016; Katz et al., 2017), it is possible that our results are mediated by baseline performance (i.e., face vs. object tasks). Albeit only a within-subjects design could clarify the issue, we wish to underline that in the current study the four groups (analysis 2) did not differ on a baseline task (CFPT) we have administered *before* the tDCS setup, and the two races did not differ in the sham condition. Thus, we suggest that the face-specific effect in non-Caucasians is genuine.

## Conclusions and future directions

Along with our previous findings (Barbieri et al., 2016) we provide evidence that albeit a-tDCS often leads to enhanced visual cognition, c-tDCS does not have an effect (neither beneficial nor detrimental). However, our prelaminar evidence suggests that c-tDCS may have a differential effect depending on the participants' race (i.e., ORE). This, if replicated, might have important implications for the neurophysiological bases of the ORE.

Future studies should replicate our ORE findings, and also test whether this effect will be seen in Caucasian participants living in non-Caucasian countries (i.e., if this effect is independent from the specific race of the participants). Furthermore, it would be of interest to ascertain in larger detail whether the face-specificity of the effect in non-Caucasians is due to an impairment of holistic and/or featural processing; this could be done by directly testing holistic mechanisms by means of well-known effects such as the face-inversion effect (Yin, 1969) and the composite-face effect (Young et al., 1987). From a methodological perspective, future research should also specifically consider variables that could affect group differences such as state dependency, motivation and baseline differences (Romei et al., 2016).

## Author contributions

AC, MT: planned the study and collected participants; FB: analyzed the data and contributed to manuscript preparation; IP and MN: planned the study and contributed to manuscript preparation; DR: planned the study and wrote the manuscript.

### Conflict of interest statement

The authors declare that the research was conducted in the absence of any commercial or financial relationships that could be construed as a potential conflict of interest.
